# Prevalence, in-hospital mortality, and factors related to neurogenic pulmonary edema after spontaneous subarachnoid hemorrhage: a systematic review and meta-analysis

**DOI:** 10.1007/s10143-023-02081-6

**Published:** 2023-07-11

**Authors:** Lei Guo, Xu Yang, Bo Yang, Guo Tang, Chunling Li

**Affiliations:** 1 Department of Neurosurgery, Sichuan Academy of Medical Sciences and Sichuan Provincial People’s Hospital, University of Electronic Science and Technology of China, Chengdu, 610072 China; 2 Department of Neurology, The Tradional Chinese Medicine Hospital of Leshan, Leshan, 614000 China; 3 Department of Neurology, Sichuan Academy of Medical Sciences and Sichuan Provincial People’s Hospital, University of Electronic Science and Technology of China, Chengdu, 610072 China; 4 Department of Emergency, West China Hospital, Sichuan University, Chengdu, 610041 Sichuan China

**Keywords:** Neurogenic pulmonary edema, Subarachnoid hemorrhage, Systematic review, Meta-analysis

## Abstract

Neurogenic pulmonary edema (NPE) is a life-threatening and severe complication in patients with spontaneous subarachnoid hemorrhage (SAH). The prevalence of NPE varies significantly across studies due to differences in case definitions, study populations, and methodologies. Therefore, a precise estimation of the prevalence and risk factors related to NPE in patients with spontaneous SAH is important for clinical decision-makers, policy providers, and researchers. We conducted a systematic search of the PubMed/Medline, Embase, Web of Science, Scopus, and Cochrane Library databases from their inception to January 2023. Thirteen studies were included in the meta-analysis, with a total of 3,429 SAH patients. The pooled global prevalence of NPE was estimated to be 13%. Out of the eight studies (*n* = 1095, 56%) that reported the number of in-hospital mortalities of NPE among patients with SAH, the pooled proportion of in-hospital deaths was 47%. Risk factors associated with NPE after spontaneous SAH included female gender, WFNS class, APACHE II score ≥ 20, IL-6 > 40 pg/mL, Hunt and Hess grade ≥ 3, elevated troponin I, elevated white blood cell count, and electrocardiographic abnormalities. Multiple studies showed a strong positive correlation between the WFNS class and NPE. In conclusion, NPE has a moderate prevalence but a high in-hospital mortality rate in patients with SAH. We identified multiple risk factors that can help identify high-risk groups of NPE in individuals with SAH. Early prediction of the onset of NPE is crucial for timely prevention and early intervention.

**Supplementary Information:**

The online version contains supplementary material available at 10.1007/s10143-023-02081-6.

## Introduction

Spontaneous subarachnoid hemorrhage (SAH) is a cerebrovascular disease associated with high morbidity and mortality [1]. Neurogenic pulmonary edema (NPE) is a severe and life-threatening complication of spontaneous SAH. Two main pathogenic mechanisms have been proposed to underlie NPE: the hemodynamic theory, which posits that an increase in pulmonary vascular pressure results from a sudden surge in catecholamines in circulation; and the theory of increased pulmonary permeability, which suggests that direct damage to the lungs is caused by the massive sympathetic discharge and cytokines released as a result of elevated intracranial pressure [2].

Despite the significant impact on mortality, the prevalence and risk factors associated with NPE in the population affected by spontaneous SAH remain poorly characterized. The population-based prevalence of NPE exhibits significant variation due to differences in case definitions, recruited populations, and study methodologies [3]. Furthermore, the available data on risk factors for NPE in individuals with SAH are limited, predominantly relying on case reports or studies with small sample sizes [4, 5]. Consequently, there is a lack of comprehensive research exploring the potential utility of these factors in accurately identifying individuals at a heightened risk of developing NPE in relation to SAH.

To our knowledge, no systematic review or meta-analysis on the prevalence, in-hospital mortality, and risk factors of NPE in SAH is available. Therefore, a reliable estimate of the prevalence, in-hospital mortality, and identification of risk factors associated with NPE in the SAH population is not only necessary to identify high-risk groups of NPE in SAH individuals, but also essential for clinical decision makers, policy formulation, and precision medicine research.

The primary aim of this systematic review and meta-analysis is to determine the overall pooled prevalence and in-hospital mortality of NPE in the SAH population. The secondary aim is to provide an overview of the risk factors associated with NPE in SAH.

## Materials and methods

### Protocol and guidance

This systematic review and meta-analysis followed the Preferred Reporting Items for Systematic Reviews and Meta-Analysis (PRISMA) 2020 guidelines [6]. The protocol was submitted and approved in the International Prospective Register of Systematic Reviews Database (PROSPERO) under the registration number CRD42023405914.

### Inclusion and exclusion criteria

We considered studies to be eligible if (1) they were randomized controlled trials (RCTs) or clinical studies reporting on the prevalence, in-hospital mortality, or risk factors of NPE in the SAH population; (2) both SAH and NPE had to be diagnosed using valid and objective methods rather than self-reported; and (3) they were published as original articles in English. We excluded studies if (1) they were designed as nonhuman studies; (2) they did not report analyses of primary data (e.g., letters, editorials, or narrative reviews); or (3) they did not provide sufficient data. When articles did not report a prevalence or mortality figure, an attempt was made to estimate it from the information provided.

### Literature search and selection

All records identified in the initial search were screened by two reviewers (Xu Yang and Bo Yang) for relevance, first by title, then by abstract, and then by the full text. Any disagreement between the reviewers was resolved by consensus by a third independent reviewer (Chunling Li). Two reviewers independently conducted a systematic search using multiple literature databases, including PubMed/Medline, Embase, Web of Science, Scopus, and the Cochrane Library, from inception to January 1st, 2023.

**Fig. 1:**
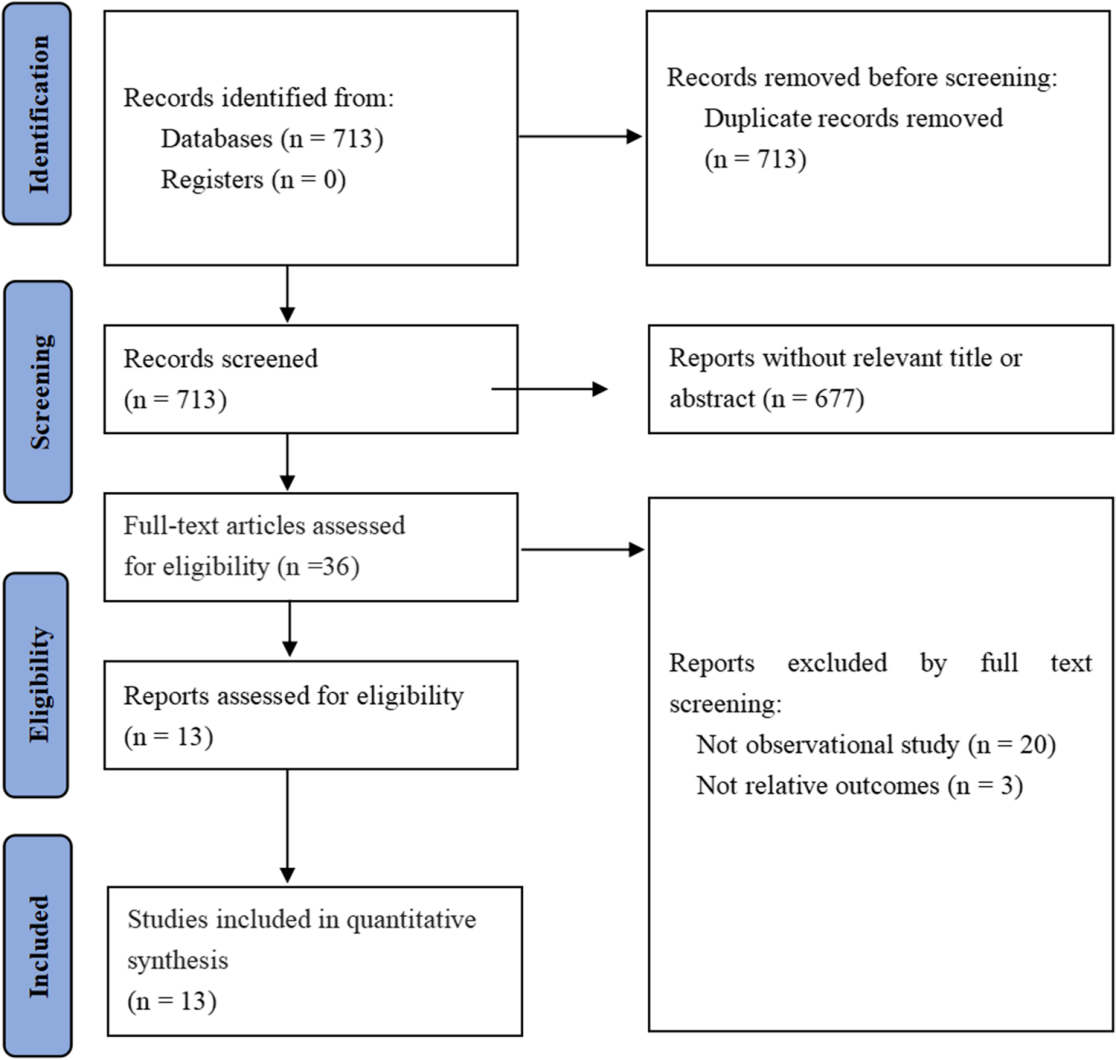
Literature search flow chart. Flowchart of the literature search and selection for systematic review and meta-analysis

Search terms were a combination of free-text terms and controlled vocabulary related to NPE and SAH. In order to define the population, "neurogenic pulmonary edema" was combined by the Boolean operator "AND" with terms that potentially evaluated subarachnoid hemorrhage, i.e., "subarachnoid hemorrhage", "stroke", "intracranial hemorrhage", and "computed tomography". We also searched ClinicalTrials.gov and the World Health Organization International Clinical Trials Registry Platform for ongoing or unpublished eligible studies. An English-language restriction was also included in our search strategy. To maximize the identification of relevant articles, we reviewed the reference lists of identified studies and contacted the authors of potentially eligible studies to obtain additional data. If multiple studies belonged to the same or partially overlapping populations, we used the data with the longest follow-up duration or the largest sample size. If duplicate studies offered results related to different outcomes, they were all included in the pooled analysis of specific outcomes. The detailed steps of the literature search are depicted in Fig. 1.

After removing duplicates, two independent researchers (Lei Guo and Guo Tang) screened all titles of studies that met the inclusion criteria, and the remaining abstracts were then screened. They then obtained full-text versions and performed further screening if studies were deemed eligible. Any disagreements were resolved by consensus.

### Data extraction

Two independent researchers (Xu Yang and Bo Yang) utilized a standardized form to extract data from the included studies. The following data were extracted from the eligible published articles: (1) first author's name; (2) year of publication; (3) study location; (4) age range and sex; (5) study sample size; (6) number of NPE cases; (7) reported risk factors of NPE; and (8) prevalence of NPE.

### Quality assessment

Two researchers (Lei Guo and Guo Tang) independently assessed the quality of the included studies using the Newcastle–Ottawa Scale (NOS; http://www.ohri.ca/programs/clinical_epidemiology/oxford.asp). The NOS checklist consists of three sections: selection, comparability, and outcome. Each section was assigned a maximum of four, two, and three points, respectively. According to NOS thresholds, 1–3 points indicated poor quality, 4–6 points indicated fair quality, and 7–9 points indicated high quality.

### Data synthesis and statistical analyses

All statistical analyses were performed using Stata software version 17.0 (StataCorp, College Station, Lakeway, TX, USA). The primary outcome of this meta-analysis was the pooled prevalence of NPE in SAH patients, which was n (%). Proportions were transformed using the Freeman-Tukey double arcsine transformation to calculate an overall proportion. We performed an inverse-variance meta-analysis with the use of a random-effects model to estimate the overall pooled effect among the studies. The extent of heterogeneity was quantified by the Q test [7] and I^2^ score [8]. If heterogeneity existed, meta-regression was performed to explore the source of heterogeneity.

Sensitivity analyses were performed to evaluate the stability of the results, by omitting each study one by one and recalculating the combined effect size on the remaining studies. Publication bias was assessed qualitatively by the visual estimation of funnel plots and quantitatively by calculating Egger’s test. We considered a P-value less than 0.05 to represent the possibility of small-study effects.

## Results

### Study selection

A total of 713 articles were identified through the database search described above. In the comprehensive literature search, relevant articles were yielded when duplicates were excluded. After removing duplicates, the comprehensive literature search yielded relevant articles. Following full-text examination, 700 records were excluded, leaving thirteen studies for inclusion (Fig. 1). Assessment of the reference lists did not yield any further eligible articles.

### Quality assessment

The quality of all included studies (cohort and case–control studies) was evaluated using the Newcastle–Ottawa Scale (NOS) score. Scores of 0–3, 4–6, and 7–9 were considered to indicate low, fair, and high quality, respectively. All included studies had NOS scores of ≥ 7, indicating a lower risk of bias and better quality (Supplementary Table 1).

### Review of publication bias

**Fig. 2:**
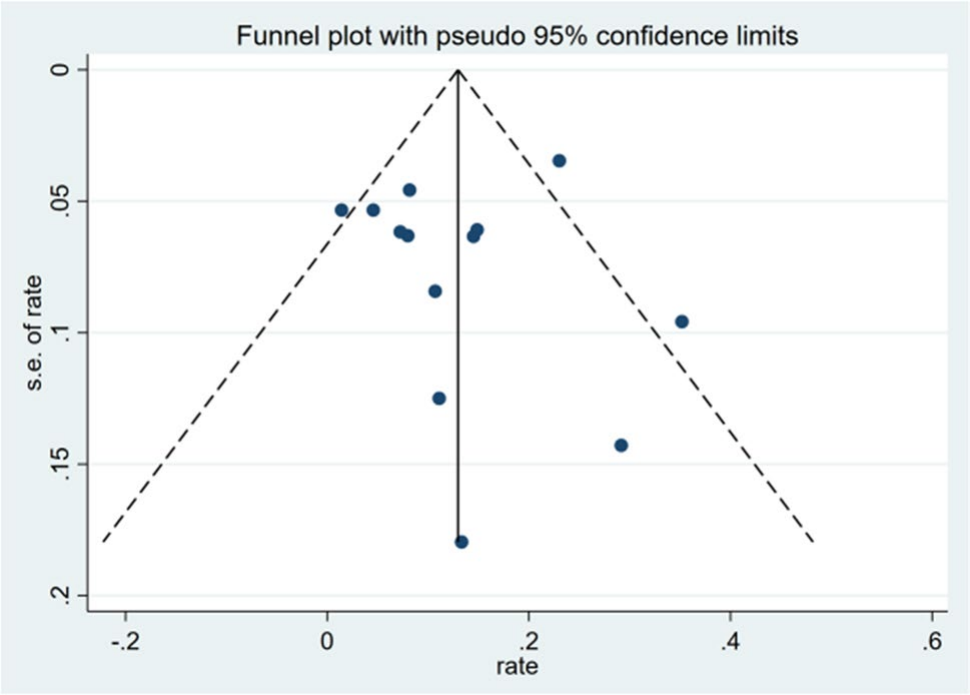
Funnel plot

A funnel plot was conducted to evaluate publication bias, and visual assessment revealed asymmetry (Fig. 2). Egger’s test showed no significant small-study effects (*P* = 0.064). Therefore, no publication bias was present among the studies included in this meta-analysis.

### Characteristics of participants

Table 1 presents the main characteristics of the thirteen studies [9–21] included in this analysis. All eligible studies were published between 2001 and 2020, with a median sample size of 264 (interquartile range: 108–350), comprising a total of 3,429 patients. For the geographic area, eight studies (62%) were conducted in Asia [9, 12, 13, 15–17, 19, 21], four (31%) were conducted in Europe [11, 14, 18, 20], and only one (7%) was conducted in the United States [10].

**Table 1:** Characteristics of total study population

First author	Publication year	Area	Age	Sex	Male(%)	Sample size	Prevalence	Quality assessment
Hidenobu OCHIAI	2001	Japan	62.98	M/F (13/35)	27.1	48	29.17%	7
Jonathan A. Friedman	2003	American	51	M/F (113/192)	37	305	1.64%	9
Carl Muroi	2008	Switzerland	54.4	M/F (338/139)	70.9	477	8.18%	7
I-Chang Su	2009	China	52	M/F (11/19)	36.7	30	13.33%	7
Joji Inamasu	2012	Japan	67.1	M/F (20/43)	31.7	63	11.11%	7
Eija Junttila	2013	Finland	58.7	M/F (56/52)	51.9	108	35.19%	9
Etsuko Satoh	2014	Japan	48	M/F (48/92)	34.3	140	10.71%	7
Wei-Lung Chen	2016	China	57	M/F (111/158)	41.3	269	14.87%	7
Wei-Lung Chen	2016	China	56	M/F (99/149)	39.9	248	14.52%	7
A. Saracen	2016	Poland	25–69	M/F (140/110)	56	250	8.00%	7
Limin Zhang	2016	China	62	M/F (346/488)	41.5	834	23.02%	7
Tijana Nastasovic	2017	Serbia	52.4	M/F (100/162)	38.2	262	7.25%	7
Tatsuki Kimura	2020	Japan	61.8	M/F (117/233)	33.4	350	4.57%	7

### Prevalence of NPE in SAH patients

**Fig. 3:**
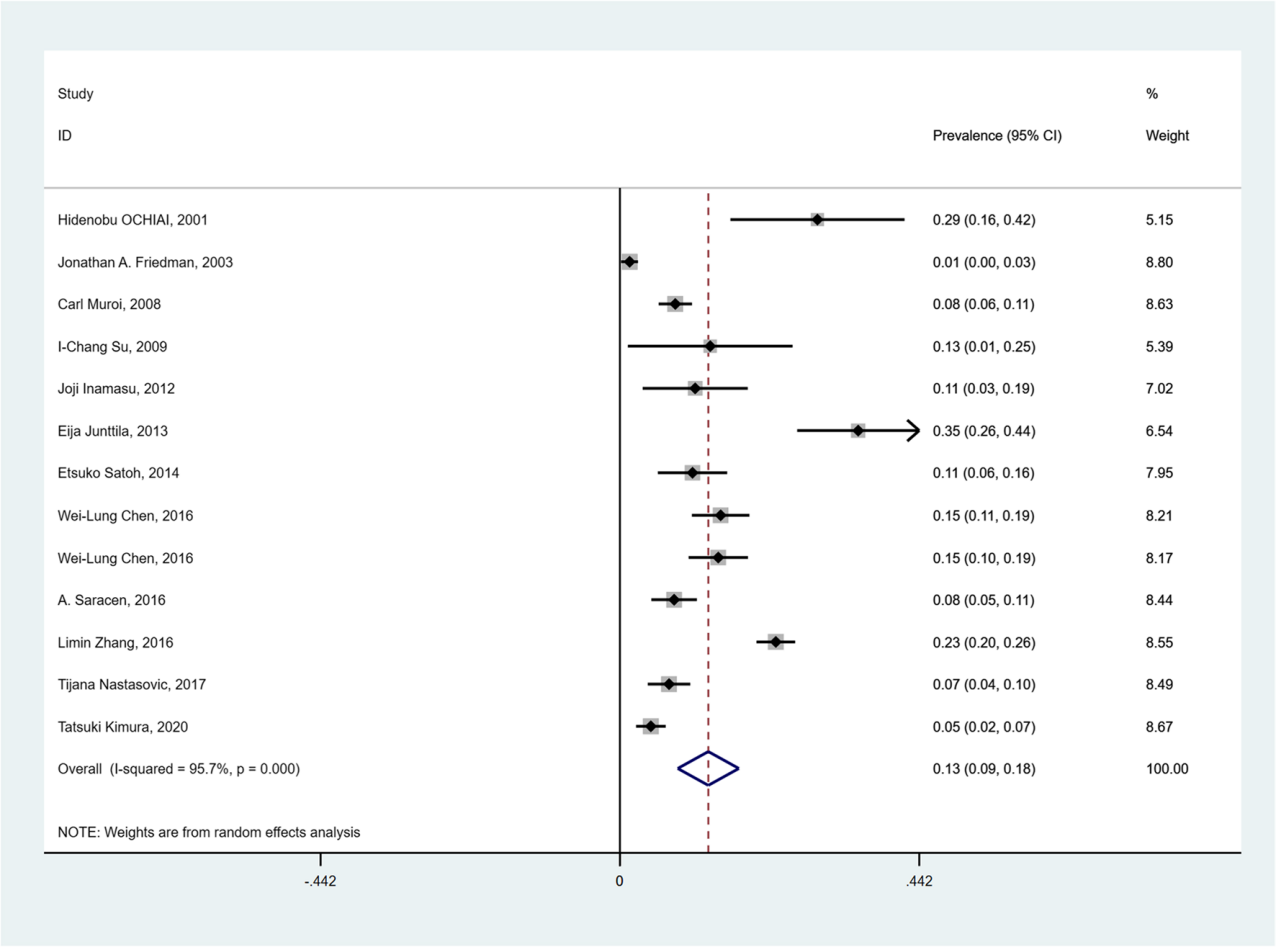
Forest plot of the prevalence of NPE in SAH patients

All thirteen studies [9–21] involving a total of 3429 SAH patients were used to calculate the overall prevalence of NPE. The overall pooled prevalence of NPE in SAH patients was 13% (95% CI = 0.09—0.18; I^2^ = 95.7%,  < 0.001, ph < 0.001) in a random-effects model (Fig. 3). There was heterogeneity among the included studies (I^2^ = 95.7%, Ph < 0.001) that reported the prevalence of NPE in SAH patients. Accordingly, meta-regression analyses were performed to explore the sources of heterogeneity. The results of meta-regression analyses did not identify any sources of heterogeneity in each indicator (Supplemental Table 1). Sensitivity analysis revealed that one study [18] might be the source of heterogeneity (Supplemental Fig. 1). When the study was removed, there was evidence of low heterogeneity in the twelve remaining studies, but the results did not change substantially (Prevalence: 11%; 95% CI = 0.07—0.16; I^2^: 0%; Ph = 0.990, *p* = 0.006). The heterogeneity may have been due to the less subjective diagnosis of NPE compared to the other twelve studies.

### Risk factors related to NPE in SAH patients

Eight studies with 2,274 patients reported risk factors related to NPE after spontaneous SAH. We categorized the identified risk factors into four main groups: severity of primary SAH, inflammatory cytokines, biomarkers associated with cardiac dysfunction, and demographic characteristics. These risk factors included World Federation of Neurological Surgeons (WFNS) class, APACHE II score ≥ 20, Hunt and Hess grade ≥ 3, IL-6 > 40 pg/mL, elevated troponin I, elevated white blood cell count, and electrocardiographic (ECG) abnormalities, and female gender (Table 2). Among them, three studies found that WFNS class was an independent risk factor for NPE [16, 17, 21].

**Table 2:** Risk factors of neurogenic pulmonary edema in subarachnoid hemorrhage

First author	Publication year	Area	Risk factors	OR	95% CI
Joji Inamasu	2012	Japan	Norepinephrine	1.003	1.002–1.007
Eija Junttila	2013	Finland	APACHE II score ≥ 20	6.17	1.30–29.21
			IL-6 > 40 pg/mL	5.62	1.26–25.08
			cTnI > 0.06 μmol/L	4.24	0.97–18.58
Etsuko Satoh	2014	Japan	Lactate level	1.04	1.00–1.09
			Age	0.9	0.83–0.97
			Glucose level	1.03	1.01–1.05
Wei-Lung Chen	2016	China	WFNS class *	5.8	2.6–13.3
			Abnormal Q or QS wave	3.1	1.1–9.1
			nonspecific ST- or T-wave changes	3	1.2–7.5
Wei-Lung Chen	2016	China	WFNS class *	4.048	1.589–10.311
			Total power(ms2)	0.995	0.992–0.998
			Normalized low-frequency component (nu)	0.993	0.910–0.958
Limin Zhang	2016	China	corrected QT prolongation	1.5	1.1–2.2
			ST depression	2.3	1.0–5.2
			nonspecific ST or T-wave change	2.7	1.8–4.2
Tijana Nastasovic	2017	Serbia	Female	5.253	1.14–24.16
			Hunt and Hess grade ≥ 3	12.593	1.27–124.79
			Hydrocephalus	8.075	1.55–42.06
			Elevated troponin I	4.862	1.26–18.74
			Elevated white blood cell count	21.867	4.02–118.75
Tatsuki Kimura	2020	Japan	WFNS grade 3–5	3.73	1.02–13.66
			VA dissection	4.83	1.50–15.56

World Federation of Neurological Surgeons (WFNS) class (class I, Glasgow Coma Scale [GCS] = 15, no motor deficit; class II, GCS = 13- 14, no motor deficit; class III, GCS = 13–14, presence of motor deficit; class IV: GCS = 7–12; class V: GCS = 3–6. * The OR for WFNS class represents the OR for each 1-point increase from low-grade WFNS (I-III) to high-grade WFNS (VI-V)

### In-hospital mortality

**Fig. 4:**
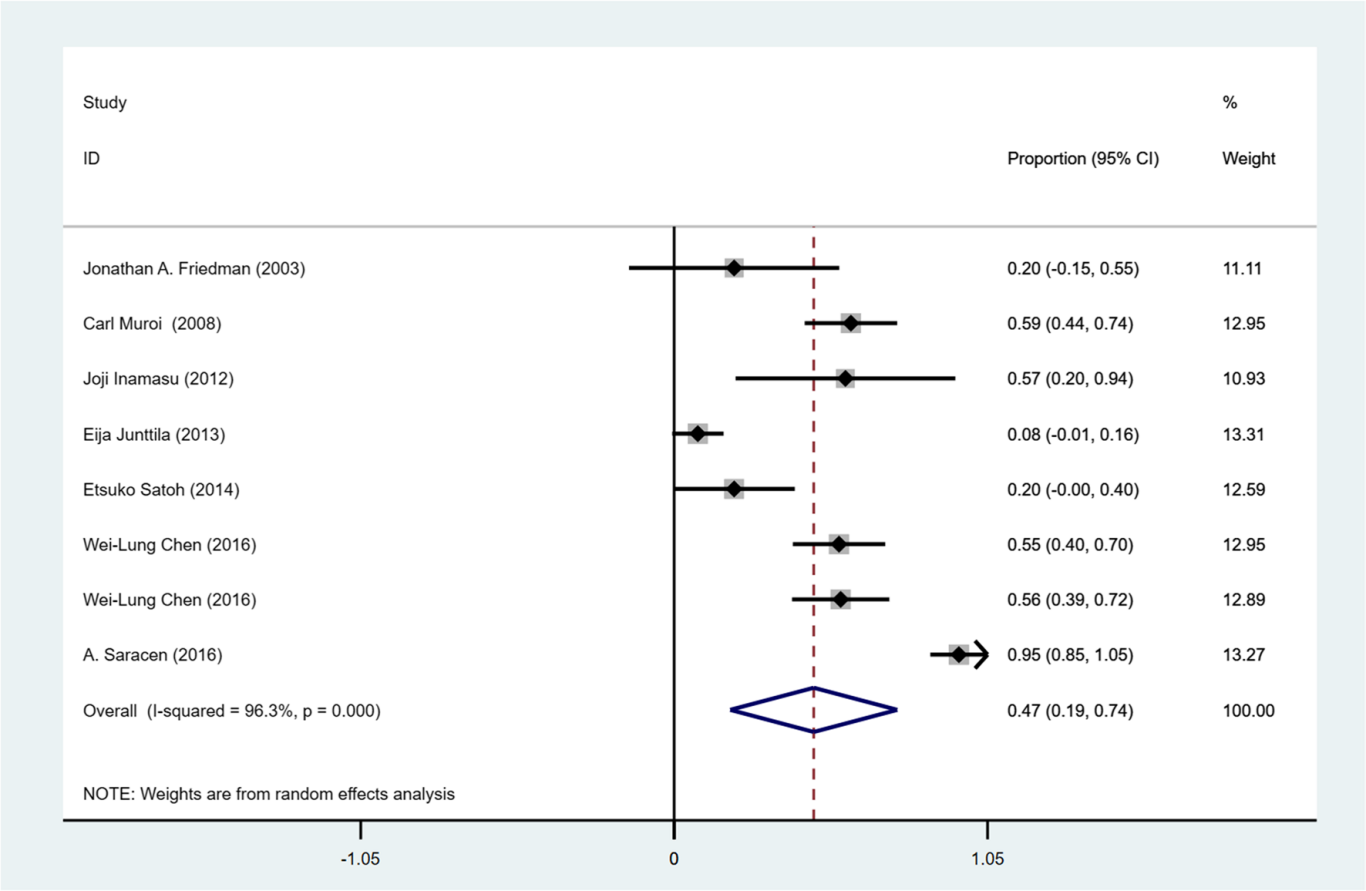
Meta-analysis of overall in-hospital mortality in NPE patients with SAH

Eight studies (*n* = 1095, 56%) reported the number of in-hospital mortality cases of NPE among patients with SAH. The pooled in-hospital death proportion was 47% (95% CI = 0.19—0.74; I2 = 96.3%; *P* = 0.001, Ph < 0.001, Fig. 4). There was substantial heterogeneity among the included studies (I^2^ = 96.3%, Ph < 0.01) reporting the in-hospital mortality of NPE in SAH patients. The results of meta-regression analyses did not identify the sources of heterogeneity in each indicator (Supplemental Table 3). The sensitivity analysis revealed that no heterogeneity source was found in the sensitivity analysis (Supplemental Fig. 2).

## Discussion

To date, no systematic effort has been made to review the epidemiological features of NPE in SAH patients in a meta-analysis. In this study, we have firstly performed a comprehensive analysis of the prevalence and in-hospital mortality that are associated with NPE in patients with SAH. Our results also highlight risk factors that can be used to identify SAH patients with a high risk of developing NPE on a more personalized basis.

Our study, which involved 3,429 adult SAH patients, demonstrated that the prevalence of NPE was up to 13%, which is well within the reported range of 2–31% [3]. The occurrence of NPE was reported to have a bimodal distribution, with the highest prevalence occurring in the hours and minutes following initial SAH and 12–24 h after SAH [22]. Clinical manifestations consist of non-specific signs of respiratory distress, such as dyspnea, tachypnea, hypoxia, pink expectoration, and crackles on auscultation, which usually resolve within 24–48 h in 50% of patients [2]. This emphasizes the important relationship between brain and lung, indicating that massive sympathetic discharge and catecholamine surges after acute SAH may be responsible for causing NPE shortly after the initial insult.

According to our results, the highest prevalence of NPE in SAH patients was reported in Finland, with a rate of 35.19% [14], while the lowest prevalence was reported in America, with a rate of 1.64% [10]. By observing the prevalence in different regions, we can conclude that the prevalence of NPE in different populations is completely different, which may be attributed to cultural differences or variations in the tools and methods used in the studies.

Spontaneous SAH is associated with significant morbidity and mortality, with reported rates ranging from 27 to 44%. While there has been a decline in SAH mortality rates over the past decades, the overall 30-day case-fatality rate for SAH is still considerable at 26.7% [23]. In our study, we found that the in-hospital mortality among SAH patients with NPE was as high as 47%, which is substantially higher than the overall mortality in SAH patients without NPE. This high mortality rate can be attributed to the severity of the underlying brain injury and respiratory dysfunction in patients with NPE [24]. Furthermore, SAH patients with NPE are more prone to medical complications, leading to longer hospital stays and intensive care unit (ICU) admissions compared to SAH patients without NPE. Common complications include bacteremia, central line infections, urinary tract infections, aspiration pneumonia, nutritional deficiencies, deep venous thrombosis, and anemia, all of which significantly impact patient outcomes [25, 26]. Therefore, early identification and prevention of NPE, as well as pulmonary interventions such as lung protective ventilation and anti-sympathetic treatment, are crucial in improving the outcome of these critical patients. Moreover, it is essential to gain a better understanding of the relationship between brain and lung in order to effectively manage NPE and its complications in SAH patients.

Given that NPE involves multiple mechanisms and many signaling pathways, various studies have identified different risk factors associated with NPE in patients after SAH. Firstly, the severity of primary SAH plays a crucial role in predicting the occurrence of NPE. The APACHE II Score, which assesses the severity of illness and prognosis in critically ill patients based on physiological parameters and clinical indicators [27], was found to be an independent predictor of NPE [14]. Additionally, the Hunt and Hess grading system and the WFNS class are widely used severity assessment tools for SAH. These grading systems categorize SAH patients based on the manifestation of neurological symptoms and the level of consciousness, and they are known to be associated with the prognosis of SAH patients [5]. In our study, we found that the Hunt and Hess grade [20] and WFNS class [16, 17, 21] were independent predictors for NPE in SAH patients, possibly due to an increased intracranial pressure and excessive activation of the sympathoadrenal axis that can lead to the formation of a reaction cascade. These findings indicate that patients with higher Hunt and Hess grades or worse WFNS class are at an increased risk of developing NPE. It highlights the importance of assessing the severity of SAH using these grading systems in identifying patients who may be more susceptible to NPE.

Norepinephrine, a catecholamine that acts as both a neurotransmitter and a hormone, has important physiological effects on the cardiovascular system. It can constrict blood vessels, increase cardiac contractility, and elevate the heart rate, which helps promote blood circulation. Additionally, norepinephrine plays a role in regulating renal blood flow and glomerular filtration rate [28]. In SAH patients complicated by NPE, a study conducted by Joji Inamasu et al. observed changes in plasma norepinephrine levels and found that norepinephrine is actively involved in this condition [13]. The study suggested that increased plasma norepinephrine levels may contribute to the development of NPE. Norepinephrine can stimulate α1-adrenoceptors, leading to increased permeability and capillary pressure in the pulmonary microvasculature [29]. It can also stimulate β1-adrenoceptors, resulting in overperfusion and edema of the lungs due to increased right ventricular systolic pressure [30]. Based on these findings, it is reasonable to assume that NPE develops more frequently in the presence of increased norepinephrine levels. To reduce the incidence of NPE, early adequate sedation, analgesia, and the suppression of sympathetic nervous system activity may be beneficial for SAH patients with increased norepinephrine levels.

Cardiac dysfunction, as indicated by elevated levels of cardiac biomarkers and abnormal electrocardiographic (ECG) findings, has emerged as a significant predictor of NPE in several studies [14, 16, 17, 19, 20]. The multifactorial nature of myocardial injury following SAH involves direct cardiac toxicity resulting from the release of norepinephrine from intracardiac nerve endings, systemic hyperadrenergic states, and coronary vasospasm [31]. Notably, cardiac troponin, a specific protein predominantly found in cardiac muscle cells, has exhibited exceptional specificity and sensitivity as a biomarker for NPE [14, 20]. Additionally, ECG changes often reflect myocardial injury. A cohort study by Chen et al. [16] demonstrated that a decrease in cardiac variability and depressed sympathovagal modulation, represented by lower total power and a normalized low-frequency component, may serve as early predictors of NPE in SAH patients. Later, Wei-Lung et al. [17] assessed if ECG abnormalities could predict the development of NPE in cases of spontaneous SAH and found that abnormal Q or QS waves and nonspecific ST or T wave changes are significant variables associated with NPE. These findings were once again confirmed in the study by Limin Zhang et al. [19]. Moreover, cardiac dysfunction can impair systemic blood flow, resulting in less oxygen being delivered to the tissue, which in turn leads to increased anaerobic metabolism and overproduction of lactate. Accordingly, an increased serum lactate level, occurring within 1 h of the ictus, is an independent factor associated with the early onset of NPE [16].

The hypothesis of a brain injury-triggered systemic inflammatory response as an underlying mechanism of NPE has gained scrutiny since the 1980s [32]. Previous research has demonstrated increased serum levels of inflammatory cytokines, including interleukin 8, growth-related genes alpha, and interleukin 6 (IL-6) in patients with NPE following non-traumatic brain injury [33, 34]. Among these cytokines, only systemic IL-6 concentration was confirmed as an independent predictor for NPE in Junttila, Eija et al.’s study [14], particularly when the IL-6 plasma concentration exceeds 40 pg/mL (normal serum levels of IL-6 typically range from 1 to 10 pg/mL). Subsequently, Nastasovic et al. [20] suggested that an elevated white blood cell count could also be used as an independent predictor of NPE in patients with SAH. Consequently, it is necessary for clinics to routinely monitor these inflammatory cytokines to identify high-risk groups of NPE. Nonetheless, further studies are required to consider the possibilities for reducing this inflammatory response.

Although aging and female sex have been identified as potential risk factors for SAH [35], current evidence does not definitively establish whether these factors are associated with an increased risk of NPE in SAH patients. In the eight studies that examined risk factors for NPE following spontaneous SAH, various demographic factors were analyzed, including age, sex, and history of hypertension. However, only two studies found that younger age [15] and female sex [20] might be potential risk factors. Further prospective studies are needed to determine whether these two factors are related and in which direction.

Notably, a recent publication by Fan et al. [36] has conducted a similar study on the prevalence and outcome of acute respiratory distress syndrome (ARDS) in traumatic brain injury (TBI), offering valuable insights into the comparison between these two conditions [36]. This study revealed noteworthy similarities and differences between the two conditions. Firstly, both respiratory dysfunctions arise from central nervous system damage, resulting in shared pathophysiological mechanisms such as activation of the sympathoadrenal axis and a systemic inflammatory response. Conversely, while the pooled prevalence of ARDS in TBI patients (19%) surpasses the prevalence of NEP in SAH patients (13%), the overall mortality rate upon discharge for TBI patients (30%) is lower compared to SAH patients with NPE (47%). However, it is important to interpret these findings with caution due to the high heterogeneity observed across the studies.

### Limitations

The strength of this study lies in the large number of patients included in the analysis, which enabled the assessment of the prevalence, in-hospital mortality, and risk factors of NPE following SAH. However, the methods employed in this study have limitations. These limitations are primarily attributable to the low quality and high heterogeneity of the individual participant data used in the meta-analysis. Although several potential confounders were identified and sensitivity analyses conducted, residual confounding remains a possibility. Furthermore, the retrospective design of most of the included studies resulted in selection bias and limited the generalizability of the findings. The single-center design of most studies also limits external validity since these centers likely have more experience in treating and operating on SAH and NPE, leading to a higher level of treatment that may positively distort outcome data. Another limitation is the inability of our study to account for variations in patient baseline comorbidities and their potential impact on mortality and the development of NPE. Finally, it is not possible to determine the utility of combining risk factors for NPE from our analysis, and one should exercise caution when pooling estimates for multiple variables.

## Conclusion

The study reveals that NPE has a moderate prevalence but a high in-hospital mortality rate in patients with SAH. The identification of clinical, ECG, and blood-based biomarkers associated with NPE after SAH suggests the potential for early recognition and timely prevention of NPE on a more personalized basis. However, more high-quality studies are urgently required to strengthen the evidence base for uncovering more promising approaches to prevent and intervene in NPE in patients with SAH.

### Supplementary Information

Below is the link to the electronic supplementary material.

Supplementary file1 (DOCX 124 KB)

## Data Availability

Data are available upon reasonable request.
