# Supplementary material for: Prevalence, in-hospital mortality, and factors related to neurogenic pulmonary edema after spontaneous subarachnoid hemorrhage: a systematic review and meta-analysis
Source: Neurosurg Rev. 2023 Jul 11;46(1):169. doi: 10.1007/s10143-023-02081-6 (PMC10335949; doi:10.1007/s10143-023-02081-6)
Supplement: Supplementary file 1 — Supplementary file1 (DOCX 124 KB) [file 10143_2023_2081_MOESM1_ESM.docx]

**Supplemental Materials**

Supplementary Table 1. Newcastle Ottawa Scale assessments......................................................................................................................................................2

Supplementary Table 2. Meta-regression analyses in prevalence of SAH patients with NPE.......................................................................................................3

Supplementary Table 3. Meta-regression analyses in-hospital mortality of SAH patients with NPE............................................................................................4

Supplemental Fig. 1 Sensitivity analysis of prevalence of NPE in SAH patients..........................................................................................................................5

Supplemental Fig. 2 Sensitivity analysis of in-hospital mortality of NPE in SAH patients...........................................................................................................6

**Supplementary Table 1. Results of quality assessment using the Newcastle-Ottawa Scale.**

| **Study** | **Selection** | | | **Comparability** | | **Outcome** | | | **Scores** |
| --- | --- | --- | --- | --- | --- | --- | --- | --- | --- |
|  | **Representativeness**  **of the exposed cohort** | **Selection of the non-exposed cohort** | **Ascertainment of exposure** | **Demonstration that outcome of interest was not present at start of study** | **Comparability of cohorts on the basis of design or analysis** | **Ascertainment of outcome** | **Was follow-up long enough for outcome** | **Adequace of follow-up of cohorts** |  |
| Hidenobu OCHIAI, 2001 | ⭐ | ⭐ | ⭐ | ⭐ | ⭐⭐ | ⭐ |  |  | 7 |
| Jonathan A. Friedman, 2003 | ⭐ | ⭐ | ⭐ | ⭐ | ⭐⭐ | ⭐ | ⭐ | ⭐ | 9 |
| Carl Muroi, 2008 | ⭐ | ⭐ | ⭐ | ⭐ | ⭐⭐ | ⭐ |  |  | 7 |
| I-Chang Su, 2009 | ⭐ | ⭐ | ⭐ | ⭐ | ⭐⭐ | ⭐ |  |  | 7 |
| Joji Inamasu, 2012 | ⭐ | ⭐ | ⭐ | ⭐ | ⭐⭐ | ⭐ |  |  | 7 |
| Eija Junttila, 2013 | ⭐ | ⭐ | ⭐ | ⭐ | ⭐⭐ | ⭐ | ⭐ | ⭐ | 9 |
| Etsuko Satoh, 2014 | ⭐ | ⭐ | ⭐ | ⭐ | ⭐⭐ | ⭐ |  |  | 7 |
| Wei-Lung Chen, 2016 | ⭐ | ⭐ | ⭐ | ⭐ | ⭐⭐ | ⭐ |  |  | 7 |
| Wei-Lung Chen, 2016 | ⭐ | ⭐ | ⭐ | ⭐ | ⭐⭐ | ⭐ |  |  | 7 |
| A. Saracen, 2016 | ⭐ | ⭐ | ⭐ | ⭐ | ⭐⭐ | ⭐ |  |  | 7 |
| Limin Zhang, 2016 | ⭐ | ⭐ | ⭐ | ⭐ | ⭐⭐ | ⭐ |  |  | 7 |
| Tijana Nastasovic, 2017 | ⭐ | ⭐ | ⭐ | ⭐ | ⭐⭐ | ⭐ |  |  | 7 |
| Tatsuki Kimura, 2020 | ⭐ | ⭐ | ⭐ | ⭐ | ⭐⭐ | ⭐ |  |  | 7 |

**Supplemental table 2. Results of meta-regression analyses in prevalence of SAH patients with NPE.**

| **Factors** | **Coefficient** | **Standard error** | **P value** |
| --- | --- | --- | --- |
| Year | 0.015306 | 0.04646 | 0.750 |
| Age | 0.069295 | 0.0449674 | 0.162 |
| Sex | 0.0175573 | 0.0238625 | 0.483 |
| Sample size | -0.0010268 | 0.001264 | 0.440 |

**Supplemental table 3. Results of meta-regression analyses of in-hospital mortality of SAH patients with NPE.**

| **Factors** | **Coefficient** | **Standard error** | **P value** |
| --- | --- | --- | --- |
| Year | 1.04981 | 0.0216976 | 0.241 |
| Age | 1.006207 | 0.0216976 | 0.290 |
| Sex | 1.018504 | 0.0133983 | 0.258 |
| Sample size | 0.9861297 | 0.0125495 | 0.353 |

**Supplemental Fig. 1 Sensitivity analysis of prevalence of NPE in SAH patients.**

**Supplemental Fig. 2 Sensitivity analysis of in-hospital mortality of NPE in SAH patients.**
